# Altered Corneal Innervation and Ocular Surface Homeostasis in FHV-1-Exposed Cats: A Preliminary Study Suggesting Metaherpetic Disease

**DOI:** 10.3389/fvets.2020.580414

**Published:** 2021-01-26

**Authors:** Lionel Sebbag, Sara M. Thomasy, Adriana Leland, Madison Mukai, Soohyun Kim, David J. Maggs

**Affiliations:** ^1^Koret School of Veterinary Medicine, Hebrew University of Jerusalem, Rehovot, Israel; ^2^Department of Surgical and Radiological Sciences, School of Veterinary Medicine, University of California, Davis, Davis, CA, United States; ^3^Department of Veterinary Clinical Sciences, Iowa State University College of Veterinary Medicine, Ames, IA, United States

**Keywords:** feline herpesvirus type 1, metaherpetic disease, lacrimal functional unit, corneal innervation, nasolacrimal reflex, confocal microscopy

## Abstract

Metaherpetic disease is recognized in humans affected by herpes simplex virus-1 but is not reported in cats affected by feline herpesvirus-1 (FHV-1) despite the high prevalence of herpetic disease in this species and strong similarities in viral biology between alphaherpesviruses of humans and cats. This preliminary work evaluated cats naïve to FHV-1 (*n* = 9 cats, 18 eyes; control population) and cats naturally exposed to FHV-1 (*n* = 4 cats, 7 eyes), as confirmed by serologic testing and review of medical records. Antemortem assessment included clinical scoring, blink rate, corneal aesthesiometry, tear film breakup time (TFBUT), and Schirmer tear test-1 (STT-1) with or without the nasolacrimal reflex. Post-mortem assessment involved confocal microscopy of the corneas and evaluation of corneal nerves with ImageJ. Groups were compared with Student's *t*-tests and results are presented as mean ± standard deviation. Compared to control, herpetic cats had significantly higher (*P* ≤ 0.010) clinical scores (0.2 ± 0.4 *vs*. 4.6 ± 2.8) and response to nasolacrimal stimulation (7.8 ± 10.8% *vs*. 104.8 ± 151.1%), significantly lower (*P* < 0.001) corneal sensitivity (2.9 ± 0.6 cm *vs*. 1.4 ± 0.9 cm), STT-1 (20.8 ± 2.6 mm/min *vs*. 10.6 ± 6.0 mm/min), TFBUT (12.1 ± 2.0 s *vs*. 7.1 ± 2.9 s), and non-significantly lower blink rate (3.0 ± 1.5 blinks/min *vs*. 2.7 ± 0.5 blinks/min; *P* = 0.751). All parameters evaluated for corneal nerves (e.g., nerve fiber length, branching, occupancy) were notably but not significantly lower in herpetic *vs*. control cats (*P* ≥ 0.268). In sum, cats exposed to FHV-1 had signs suggestive of corneal hypoesthesia and quantitative/qualitative tear film deficiencies when compared to cats naïve to the virus. It is possible these are signs of metaherpetic disease as reported in other species.

## Introduction

Corneal nerves play a critical role in maintaining ocular surface health and homeostasis, providing sensory stimuli for ocular protection (blinking) and lubrication (tear secretion), as well as trophic factors to promote epithelial integrity and corneal wound healing (1, 2). Thus, disruption of corneal nerves often leads to serious detrimental effects such as aqueous tear deficiency and neurotrophic keratopathy (3, 4). Several local and systemic etiologies can compromise corneal innervation in humans and veterinary species, including diabetes mellitus, dry eye, surgery, and ocular infections (1, 5–7). The latter is particularly well-recognized for viruses belonging to the *Herpesviridae* family given their neurotropic nature and lifelong persistence in the sensory neurons, with herpes simplex virus-1 (HSV-1) disrupting corneal innervation in humans (8–11) and canine herpesvirus-1 (CHV-1) recently associated with corneal hypoesthesia in a dog with protracted corneal disease (12). However, to the best of our knowledge, no published information exists for cats affected with feline herpesvirus-1 (FHV-1) despite the high (over 90%) prevalence of this infectious agent in the general feline population (13).

FHV-1 is a leading cause of ocular disease in cats (13). Disease pathogenesis, diagnosis, and treatment are well-described (13–15), however, the potential interplay between the virus and the feline lacrimal functional unit has not been studied to date. Uhl et al. recently described the clinical features of aqueous tear deficiency in cats, reflecting on herpesvirus-related neuropathy as a potential etiology for dry eye given the lack of therapeutic response to immunomodulatory drugs; however, the association with FHV-1 and corneal innervation was not specifically investigated in that retrospective case series (16).

The objective of this preliminary study was to compare corneal innervation and ocular surface homeostasis in cats naïve to or seropositive for FHV-1 exposure. Compared to cats naïve to FHV-1, we hypothesized that cats naturally exposed to the virus would have reduced corneal innervation, as determined by corneal aesthesiometry and confocal microscopy, and would demonstrate clinical evidence of dysfunction of the lacrimal functional unit. For the latter, the nasolacrimal reflex was performed in addition to conventional tear film diagnostic tests (e.g., quantity and quality of tears), providing valuable information on the neural pathway of lacrimation (3, 17).

## Materials and Methods

### Animals

Study subjects were recruited from cats scheduled to be euthanized for reasons unrelated to the study. Written consent was obtained from owners or caretakers, and the study was approved by the University of California's Institutional Animal Care and Use Committee (protocol #18463). All were domestic shorthair cats, and were considered in two groups. The control population consisted of 9 cats (18 eyes) naïve to FHV-1, maintained in a specific pathogen-free facility, lacking historical, or active clinical signs consistent with herpesvirus infection, and confirmed negative to FHV-1 by serologic testing for FHV-1 IgG with ELISA (18). The study population comprised 4 cats (7 eyes) with historical or active clinical signs consistent with herpetic disease, recruited from patients of the University of California, Davis William Pritchard Veterinary Medical Teaching Hospital, and confirmed to be exposed to FHV-1 based on serologic testing for FHV-1 IgG. The exact duration of exposure to FHV-1 is unknown although it is likely similar to the age of each patient since cats are typically infected with this virus during kittenhood (13).

### Clinical Testing

The following procedures were performed in each eye, allowing ≥10 min between tear tests to ensure the tear film had fully replenished (19):

• **Blink rate**: Cats were observed from a distance by two examiners (LS, AL) in a quiet environment, counting the number of complete eyelid closures in a 5-min period. The average of both counts was divided by 5, and blink rate recorded as blinks/min.

• **Clinical scoring**: Clinical assessment was conducted by a trained examiner (LS) following criteria described in a previous publication (20); briefly, the total clinical disease score (ranging from 0 to 24) was defined as the sum of all ocular (conjunctivitis, blepharospasm, ocular discharge) and non-ocular (sneezing, nasal discharge) scores.

• **Corneal sensitivity**: A Cochet-Bonnet aesthesiometer (Luneau Ophtalmologie, Chartres, France) with a 0.12-mm diameter monofilament was used to assess central corneal sensitivity in each eye as previously described (17), recording the corneal tactile sensation (CTS) as the monofilament length (in cm) that elicited consistent blink reflex in at least three out of five attempts.

• **Tear film breakup time (TFBUT)**: A modified fluorescein-impregnated paper strip (DET, Akorn Inc, Lake Forest, Ill.) was used to deliver fluorescein to the dorsal bulbar conjunctiva, and TFBUT was recorded to the nearest tenth of a second as previously described (21).

• **Schirmer tear test-1 (STT-1)**: A standard Schirmer strip (Merck Animal Health) was inserted in the lateral lower conjunctival fornix for 60 s, and tear production was recorded in mm/min (21, 22).

• **Schirmer tear test-1 with nasolacrimal stimulation (NL-STT1)**: A Schirmer strip was placed in the lateral lower conjunctival fornix of each eye, followed immediately by placing a cotton ball soaked with 70% alcohol in front of (by not touching) the animal's nostrils (17). Olfactory stimulation was sustained throughout the Schirmer testing (1 min), and results were recorded in mm/min.

### Confocal Microscopy

**Figure 1:**
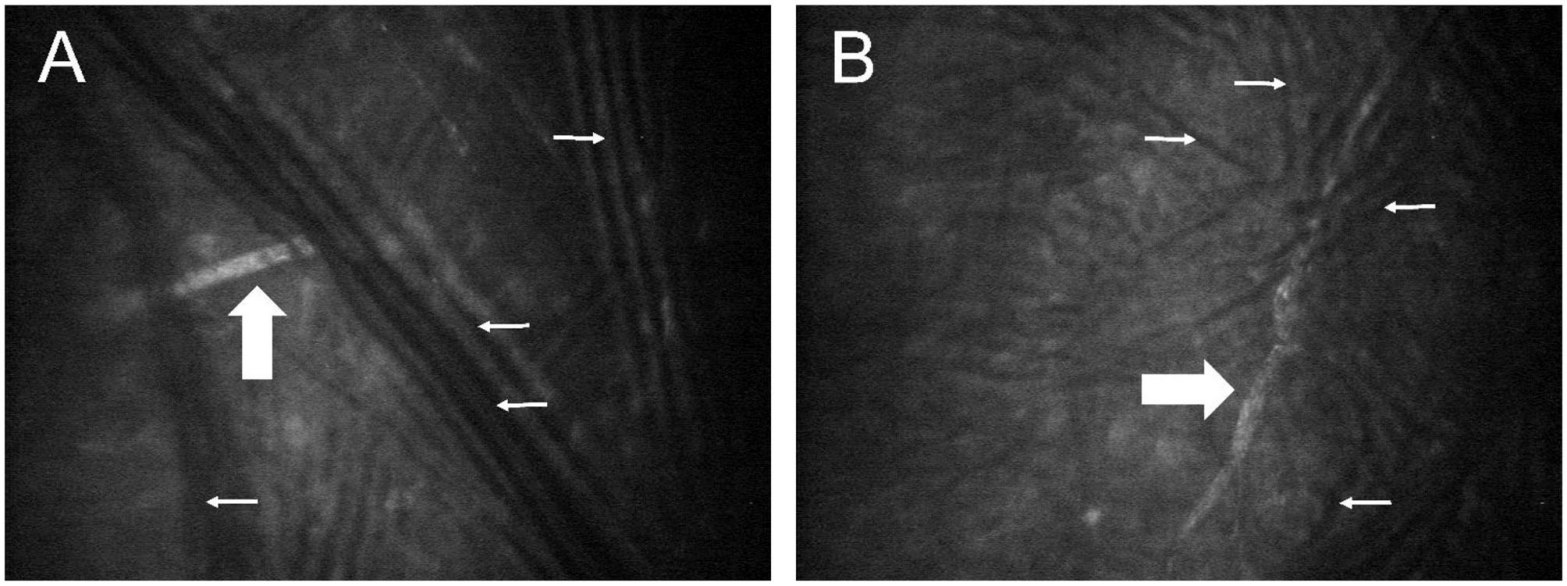
Post-mortem confocal microscopy images of feline corneas, obtained during technique validation by approaching a freshly enucleated globe **(A)** or a globe *in situ* **(B)** with the confocal probe. Multiple dark lines observed in both images (small white arrows) represent fold artifacts that interfered with assessment of corneal stromal nerves (broad white arrows).

Immediately following euthanasia, the ocular surface of both eyes was protected with generous lubrication (1% sodium hyaluronate; Hylartin V, Pharmacia & Upjohn Co) and a temporary tarsorrhaphy performed with skin staples. In a pilot study, appropriate confocal microscopy technique (ConfoScan4 device; Nidek, Inc.) was refined using 4 globes from 2 cats (not included in the animals mentioned above or data analysis). For the first cat, freshly enucleated globes were held in front of the imaging unit; for the second cat, imaging was performed with the globes *in situ*, but without third eyelid retraction. In both cases, notable artifacts precluded proper visualization of corneal nerves (Figure 1). Therefore, for study cases (*n* = 10 cats), non-enucleated feline globes were imaged *in situ* within 1 h of euthanasia, using an assistant to retract the nictitating membrane with a cotton-tipped applicator. The latter was critical in improving access to the central cornea and reduce folding artifacts presumed to be due to post-mortem ocular hypotension. The examiner slowly advanced the cat's head toward the probe until the central cornea contacted the gel (GenTeal® gel, Novartis ophthalmics, East Hanover, NJ) on the objective lens with a working distance of ~2 mm. For each eye, a minimum of two full-thickness scans were obtained at 3-μm increments over an examination period of 5–10 min.

### Evaluation of Corneal Nerves

**Figure 2:**
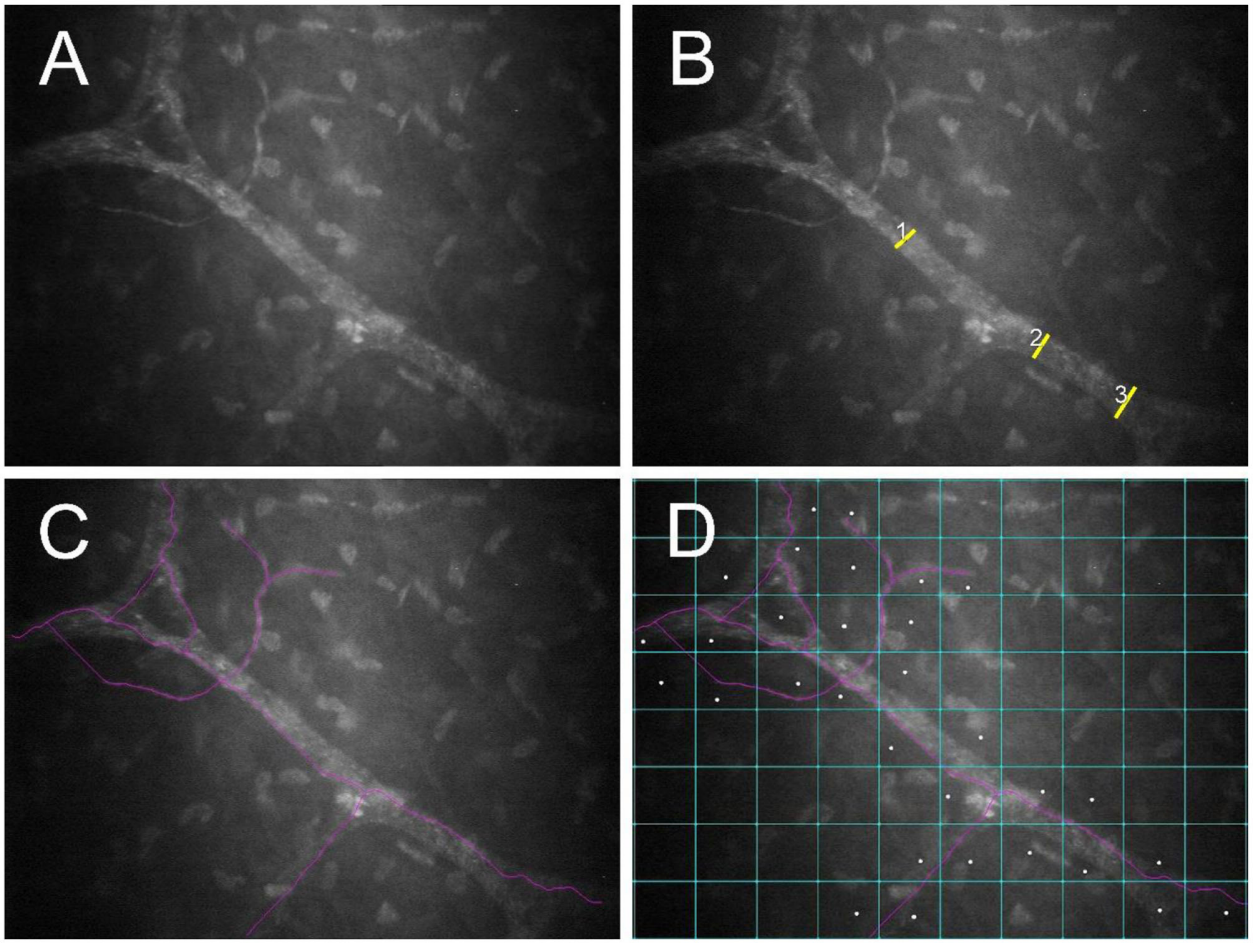
Post-mortem confocal microscopic images collected within 1 h of euthanasia of a FHV-1-naïve cat, with globes *in situ* and the third eyelid retracted using a cotton-tipped applicator. An unmodified image **(A)** as well as images demonstrating measurement of nerve thickness **(B)**, nerve fiber length **(C)**, and nerve occupancy **(D)** are shown. Images were assessed using NeuronJ software.

Corneal nerves were classified as belonging to the “sub-basal nerve plexus” if they were noted in frames immediately adjacent to the corneal epithelial basement membrane. These nerves are fine and often have an anastomotic appearance (23). By contrast, corneal nerves were classified as “stromal” if they were more distant from the epithelial basement membrane, surrounded by a dark background with bright keratocytes. These nerves characteristically follow an almost straight course and divide in a dichotomous pattern (Figure 2A) (23). Nerves were evaluated using NeuronJ, a plug-in program of ImageJ (ImageJ V.1.31) that facilitates tracing and semi-manual quantification of corneal nerves (24). A single masked observer (MM) assessed 7 parameters in each image to analyze corneal nerves, and results of 3 randomly selected images per eye were averaged:

• **Main nerve trunks**: Defined as the total number of main nerves in one image (9).

• **Nerve branching**: Defined as the total number of nerve branches (from the main trunk) in one image (9).

• **Nerve thickness**: Calculated by averaging 3 measurements of nerve fiber diameters at different points along the nerve paths (Figure 2B) (25).

• **Nerve fiber length (NFL)**: Calculated as the total length of all nerve fibers divided by the size of the frame (in μm/mm^2^), assuming a calibration factor of 0.56 μm/pixel and a frame of 0.1587 mm^2^ in scans of ConfoScan4 (Figure 2C) (26).

• **Nerve occupancy**: Calculated as the percentage of boxes in a 10 × 8 grid that contained one or more nerves (Figure 2D) (27).

• **Nerve reflectivity**: Brightness (“reflectivity”) of the nerves with respect to the background (i.e., keratocytes, collagen) was graded (0 to 4) according to criteria previously described (23).

• **Nerve tortuosity**: The tortuosity of nerves was graded (0 to 4) according to criteria previously described (23).

### Statistical Analysis

Normality of the data was evaluated with the Shapiro-Wilk test. Results are presented as mean ± SD (range) as all data were normally distributed (*P* ≥ 0.067). Student's *t*-tests were used to compare the age, antemortem clinical data, and post-mortem confocal microscopy data between the two populations. The relationship between FHV-1 IgG titers and associated clinical or confocal results was assessed using Pearson's correlation test. Statistical analysis was performed using SigmaPlot 14.0 (Systat Software Inc.), and *P* values < 0.05 were considered statistically significant.

## Results

No cat in the control population had detectable serum FHV-1 IgG whereas titers ranged from 1:320 to 1:5120 in FHV-1-exposed cats. Age of cats naïve to FHV-1 (8.6 ± 3.5 years, 4–13 years) and cats exposed to the virus (11.8 ± 4.6 years, 5–15 years) were not significantly different (*P* = 0.195). Table 1 summarizes antemortem clinical data obtained from both groups. Compared to cats naïve to FHV-1, cats exposed to the virus had significantly higher (*P* ≤ 0.010) clinical disease score and response to nasolacrimal stimulation, and significantly lower (*P* < 0.001) corneal sensitivity (CTS), tear production (STT-1), and tear film stability (TFBUT); blink rate did not differ significantly between the two populations (*P* = 0.751). One cat in the herpetic group was notably younger (5 years old) than the 3 other cats in this group (13–15 years old). Interestingly, results of STT-1 (19–20 mm/min), TFBUT (10–12 s), and corneal sensitivity (CTS 2.5 cm in both eyes) in this cat were normal.

**Table 1:** Mean ± standard deviation (range) clinical parameters associated with ocular surface health and lacrimal function in cats exposed to FHV-1 and in a FHV-1-naïve control group.

	**Cats naïve to FHV-1**	**Cats exposed to FHV-1**	***P*-value**
Clinical disease score (graded 0-24)	0.2 ± 0.4 (0–1)	4.6 ± 2.8 (1–10)	<0.001
Blink rate (blinks/min)	3.0 ± 1.5 (1–6)	2.7 ± 0.5 (2–3)	0.751
CTS (cm)	2.9 ± 0.6 (2–4)	1.4 ± 0.9 (0–2.5)	<0.001
TFBUT (s)	12.1 ± 2.0 (7.5–15.5)	7.1 ± 2.9 (4.0–12.0)	<0.001
STT-1 (mm/min)	20.8 ± 2.6 (16–25)	10.6 ± 6.0 (1–19)	<0.001
STT change with stimulation of the nasolacrimal reflex (%)	7.8 ± 10.8 (−5.3–37.5)	104.8 ± 151.1 (0–400)	0.010

*P-values (Student's t-test) are considered significant if <0.05. FHV-1, Feline herpesvirus type 1; CTS, Corneal tactile sensation; TFBUT, Tear film breakup time; STT-1, Schirmer tear test-1*.

Confocal microscopy images were available for 7 FHV-1-naïve cats (14 eyes) and 2 FHV-1-exposed cats (3 eyes). Stored images for other study subjects were lost due to a computer software failure. Although 316 confocal microscopy images were of sufficient quality to evaluate corneal nerves, the sub-basal nerve plexus could be visualized in only 9 images harvested from 4 eyes, and these data were therefore not analyzed. Considering corneal stromal nerves, no significant difference in confocal microscopic parameters was detected between cats exposed to FHV-1 and cats naïve to the virus (Table 2; *P* ≥ 0.268). However, all parameters were notably lower in herpetic *vs*. control cats.

**Table 2:** Mean ± standard deviation (range) confocal microscopic features of corneal stromal nerves of cats exposed to FHV-1 and a FHV-1-naïve control group.

	**Cats naïve to FHV-1**	**Cats exposed to FHV-1**	***P-*value**
Main nerve trunks (number trunks/image)	1.2 ± 0.3 (1–2)	1.0 ± 0 (1–1)	0.434
Nerve branching (number branches off main trunk/image)	2.3 ± 1.6 (0.8–5.2)	1.7 ± 0.9 (1–2.3)	0.648
Nerve thickness (μm)	25.2 ± 9.5 (14.6–39.1)	15.0 ± 8.9 (8.7–21.4)	0.295
Nerve fiber length (μm/mm^2^)	4,643 ± 1,733 (2,457–7,790)	3,982 ± 1,039 (3,193–4,662)	0.601
Nerve occupancy (% boxes containing ≥ 1 nerve)	22.5 ± 6.2 (14.2–32.1)	16.7 ± 5.9 (12.5–20.8)	0.268
Nerve reflectivity (graded 0–4)	2.5 ± 1.1 (1.2–4.0)	1.8 ± 2.6 (0–3.7)	0.526
Nerve tortuosity (graded 0–4)	1.6 ± 1.1 (0.7–3.7)	1.1 ± 0.6 (0.7–1.5)	0.526

*All globes were assessed in situ but post-mortem using confocal microscopy, and images were analyzed using NeuronJ software. See Materials and Methods for more details on analysis. P-values (Student's t-tests) are considered significant if <0.05. FHV-1, Feline herpesvirus type 1*.

No significant relationship was detected between FHV-1 IgG titers and associated clinical or confocal results (*P* ≥ 0.375). For non-significant results, a *post-hoc* sample size calculation (*t*-test, α = 0.05, power = 80%) showed that the minimum number of eyes required to detect significant differences between cats naïve to FHV-1 and cats naturally exposed to FHV-1 were as follows: *n* = 176 (blink rate), *n* = 111 (nerve reflectivity), *n* = 71 (nerve fiber length), *n* = 70 (nerve branching), *n* = 47 (nerve tortuosity), *n* = 37 (main nerve trunks), *n* = 19 (nerve occupancy), and *n* = 14 (nerve thickness).

## Discussion

The present study evaluated various parameters responsible for or reflective of ocular surface homeostasis in cats with or without exposure to FHV-1, and provides clinically relevant information for veterinarians managing cats infected with FHV-1. These data also may be useful for comparative research into other species affected by their host-specific *Alphaherpesvirinae*. Indeed, cats can serve as a valuable translational model given the high prevalence of spontaneously-occurring herpetic disease in feline patients, and striking similarities in viral biology and disease pathogenesis among species with natural virus-host interactions (28). In the present study, we showed that cats exposed to FHV-1 had marked and significant disturbances in the lacrimal functional unit including quantitative and qualitative tear film deficiency, reduced blink rate, and reduced corneal sensitivity, which individually and collectively are likely to be clinically important.

Quantitative tear film deficiency in herpetic cats of the present study was substantial and common. Not only was mean aqueous tear production ~2-fold lower in cats exposed to FHV-1 than in cats naïve to the virus, the majority of affected eyes had STT-1 values below the lower reference limit (9 mm/min) (21, 22). Meanwhile, all eyes of the control population had STT-1 values above 16 mm/min. These findings are similar to those reported for humans infected with HSV-1 (29). Although dry eye disease has been recognized in cats affected by various ocular surface conditions (3, 16), a potential association with FHV-1 has not been established. Evidence in the present study that stimulation of the nasal mucosa via the nasolacrimal reflex significantly increased aqueous tear production in FHV-1-exposed but not in naïve cats is similar to data from humans with latent HSV-1 infection (30). This finding is likely explained by an afferent defect in the neural pathway of lacrimation possibly due to herpesvirus-induced corneal hypoesthesia, and is supported by corneal aesthesiometry data in our study population. This highlights the importance of assessing the nasolacrimal reflex for clinical and diagnostic purposes (3, 17, 31).

Tear quality was also reduced in FHV-1-exposed cats of the current report, as has been demonstrated in humans affected with HSV-1 (29, 32). Mean TFBUT in the present study population (7.1 s) was below the lower feline reference limit (9.1 s) (21), and ~40% lower than in the control population. The underlying etiopathogenesis for this is likely multifactorial, but presumably includes reduced conjunctival goblet cells density—known to occur as early as 7 days and persisting for at least 29 days after experimental FHV-1 inoculation in cats (33)—as well as alterations in corneal epithelial cells that may affect pre-corneal ocular mucins (32).

Corneal sensitivity was markedly reduced in cats exposed to FHV-1 in the present study as evident by antemortem Cochet–Bonnet aesthesiometry. This was also supported by post-mortem assessment of corneal nerves using confocal microscopy, in which all assessed neurologic measurements were notably reduced in FHV-1-exposed cats than in the FHV-1-naïve control group. Although these differences were not statistically different, for all assessments except nerve fiber length and reflectivity, the upper limit of the data range for FHV-1-exposed cats was lower than the mean value for the control group; and for nerve fiber length and reflectivity it was very close. Failure to detect statistically significant microscopic evidence of altered corneal nerve morphology despite clinical evidence of corneal nerve dysfunction in the present study may represent the relatively low number of cats available for assessment with confocal microscopy, the sensitivity of this technique, or the fact that functional changes precede or exceed morphological changes.

To the authors' knowledge, the present work represents the first report of postmortem corneal confocal microscopy in cats, thus we first needed to optimize the technique in 4 feline eyes. Our major goal was to obtain confocal images devoid of artifacts such as corneal folds (34). Interestingly, the suggested cause of corneal folds in Jester's study was pooling of water around the stromal lamellae (34) while the present study suggests ocular hypotony as a potential factor since the so called “ridges” were no longer detectable when external pressure was applied to the globe with a cotton-tipped applicator. The advantage of post-mortem confocal microscopy is the ability to image the cornea in precise locations while avoiding micro-saccadic movements (35). However, corneal nerves can rapidly deteriorate post-mortem and affect quantitative measurements, particularly for nerves in the sub-basal plexus (35). Sub-basal nerves are reportedly degenerated by 13.5 h in human corneas (8). It is possible that similar degenerative changes occur more rapidly in cats since we were not able to detect sub-basal nerves in most feline globes despite ≤1 h elapsing between euthanasia and confocal microscopy in the present study. Discrepancy between human and feline studies may also be related to maximal resolution of the confocal device used, as well as species differences in corneal anatomy; for instance, the sub-basal nerves in humans is intimately associated with Bowman's layer (35) which is absent in feline corneas.

Three major pathophysiologic mechanisms have been used to categorize herpetic disease in humans: cytolytic, immunopathologic, and metaherpetic disease (36). The first two mechanisms are well-described for FHV-1 and explain common herpetic disease syndromes such as conjunctivitis, dendritic corneal ulcers, stromal keratitis, and eosinophilic keratoconjunctivitis seen in cats infected with FHV-1 (13). However, metaherpetic disease in cats has not been as well-described. Metaherpetic disease develops as a result of structural tissue damage subsequent to cytolytic or immunopathologic disease, and is generally non-responsive to traditional antiviral therapies because viral replication is no longer occurring. It is possible that metaherpetic disease takes time to develop and affects ocular surface homeostasis in older cats more commonly or more seriously than it does in younger subjects, as suggested by normal clinical test results in the 5-year-old cat exposed to FHV-1 in the present study. Virally-induced pathology in the central nervous system of mice inoculated with HSV-1 includes astrocyte loss and demyelination (37, 38), and the same may be true in companion animals with spontaneous herpetic disease. Corneal disease subsequent to neurologic lesions may occur due to loss of axonal transport of trophic factors (e.g., brain-derived neurotrophic factor) (39), as well as reduced tear production due to disruption of the neuronal circuitry essential for lacrimation, i.e., neurotrophic keratitis (29). Histopathological confirmation of neural pathology consistent with metaherpetic disease, especially that occurring within the central nervous system of cats in the present study would have been interesting but was not possible due to logistical and costs constraints. Regardless, the presumptive diagnosis of metaherpetic disease is strongly supported by similarities between the present findings and reported changes in other species (9, 12, 29).

The low number of cats in the study population (i.e., cats naturally exposed to FHV-1, but euthanized for reasons unrelated to the study) arose as a result of several overlapping logistical reasons: (1) several potential subjects underwent euthanasia at a local veterinary practice or other place off-site from the University, (2) investigators or equipment were not available at very short notice in the immediate antemortem and postmortem periods when specialized testing was needed, (3) some owners were unwilling to provide necessary consent.

In conclusion, the present study showed that cats exposed to FHV-1 have reduced corneal sensitivity and quantitative and qualitative tear film deficiencies when compared to cats naïve to the virus. We propose that these findings may reflect a form of metaherpetic disease associated with FHV-1 infection. Further research is required to better understand the prevalence and pathogenesis of metaherpetic disease in cats, and provide relevant information for clinicians managing FHV-1 in practice as well as scientists working with spontaneous animal models of herpesvirus infections.

## Data Availability Statement

The raw data supporting the conclusions of this article will be made available by the authors, without undue reservation.

## Ethics Statement

The animal study was reviewed and approved by the University of California's Institutional Animal Care and Use Committee (protocol #18463). Written informed consent was obtained from the owners for the participation of their animals in this study.

## Author Contributions

LS and DM conceptualized and designed the study in consultation with ST. LS, ST, and AL performed the experiments. LS, MM, and SK analyzed the data. All authors wrote the manuscript.

## Conflict of Interest

The authors declare that the research was conducted in the absence of any commercial or financial relationships that could be construed as a potential conflict of interest.
